# Exercício Físico e Teste de Caminhada de 6-min na Doença Arterial Obstrutiva de Membros Inferiores

**DOI:** 10.36660/abc.20200068

**Published:** 2020-04-06

**Authors:** Tales de Carvalho

**Affiliations:** 1 Clínica de Prevenção e Reabilitação CardiosportFlorianópolisSCBrasilClínica de Prevenção e Reabilitação Cardiosport, Florianópolis, SC – Brasil; 2 Universidade do Estado de Santa CatarinaFlorianópolisSCBrasilUniversidade do Estado de Santa Catarina (UDESC-SC), Florianópolis, SC – Brasil

**Keywords:** Doença Arterial Periférica, Claudicação Intermitente, Limiar Anaeróbio, Exercício, Teste de Caminhada, Atividades Físicas da Vida Diária

O artigo “Intensidade de Exercício durante o Teste de Caminhada de 6 Minutos em Pacientes com Doença Arterial Periférica”,^1^ com originalidade, produz informações de grande utilidade prática, a serem consideradas no diagnóstico, prognóstico e principalmente na avaliação funcional, que possibilitam um aprimoramento da prescrição de exercícios físicos no tratamento clínico da doença. O estudo foi realizado com o objetivo de determinar em pacientes sintomáticos com Doença Arterial Obstrutiva (DAO) de Membros Inferiores (MMII) se a caminhada no solo permite a detecção do primeiro limiar ventilatório, também denominado limiar anaeróbio (LA). O LA é um marcador reconhecido de intensidade do exercício, útil para a delimitação da zona considerada ideal para o treinamento físico visando o aprimoramento cardiorrespiratório.^2^

A DAO de MMII é um grande problema de saúde pública, que de acordo com o relatório global de epidemiologia, acometia 202 milhões de indivíduos em 2010, crescendo 22% até 2015, quando atingiu 237 milhões de indivíduos.^3^A associação da DAO com grandes eventos cardiovasculares (MACE) está bem documentada; em seu estágio mais grave, com isquemia crítica, apresenta elevado risco de eventos cardiovasculares, amputação de MMII e morte,^4^ associando-se a níveis elevados de troponina cardíaca e peptídeo natriurético cerebral pro-N-terminal (NT proBNP).^5^

Há forte suspeita de DAO de MMII quando ocorre dor em MMII ao esforço sem aparente etiologia ortopédica e o índice tornozelo-braquial (ITB) é menor que 0,90 em repouso. Os testes de esforço realizados com caminhadas devem ser realizados para auxiliar no diagnóstico, especialmente quando o ITB for maior que 0,91, sendo úteis também para classificação funcional e prescrição de exercícios.^6^ Os testes de campo possibilitam o diagnóstico de claudicação intermitente, com determinação das distâncias percorridas para o início da sintomatologia (claudicação inicial) e para o surgimento da máxima limitação funcional (claudicação absoluta), quando o indivíduo é obrigado a interromper a caminhada. Nos testes em esteira tem sido proposta a inclusão da medida do ITB em repouso e após exercício. A presença da doença é fortemente sugerida quando ocorre redução do ITB pós-exercício superior a 20% e a 30mmHg em relação ao observado ao repouso.^7^ O ITB em repouso, que tem sido amplamente utilizado na prática clínica, pode com certa frequência produzir um resultado falso negativo, exigindo atenção a grupos com artérias não compressíveis. No estudo de Tóth-Vajna et al.,^8^ quase um quarto dos indivíduos com diagnóstico apresentou artéria não compressível ou foi considerado sintomático com ITB negativo. Portanto, se houver suspeita clínica de DAO dos MMII, apesar dos valores normais do ITB, uma avaliação mais aprofundada deve ser considerada.

Em pacientes sintomáticos os exercícios físicos têm potencial para influir na morbidade e mortalidade, proporcionando redução dos sintomas, melhora da qualidade de vida e aumento da distância máxima caminhada, devendo obrigatoriamente integrar o tratamento otimizado.^6,7^Portanto, todos os pacientes com claudicação intermitente devem receber tratamento clínico otimizado, ou seja, uma associação de mudanças de estilo de vida com tratamento farmacológico, tendo em vista as evidências que demonstram redução de eventos cardiovasculares e melhora de desfechos relacionados aos MMII.^9^

O treinamento físico tem se mostrado seguro, sendo os exercícios realizados por meio de caminhadas com indução do sintoma de claudicação a melhor opção.^6,7^ Mas, quando a caminhada não puder ser realizada, outros tipos de exercícios, como ciclismo, exercícios resistidos e ergômetro de membros superiores, têm se mostrado efetivos.^6,7^ Cabe ainda ressaltar que exercícios físicos não podem ser realizados por pacientes com isquemia crítica, mas devem ser considerados o mais breve possível após tratamento intervencionista realizado com sucesso.^10,11^

Muitos ensaios clínicos demonstraram consistentemente que o exercício supervisionado em esteira melhora o desempenho da marcha em pessoas com DAO de MMII. A metanálise de Fakhry et al.,^12^ que avaliou 1054 indivíduos em 25 estudos, concluiu que esse tipo de exercício proporcionou em média aumento de 180 metros na distância máxima de caminhada na esteira e de 128 metros na distância caminhada sem dor.^12^

Três ensaios clínicos randomizados, que avaliaram um total de 493 pacientes com DAO de MMII, demonstraram que as intervenções com exercícios baseados em domicílio, que incorporaram técnicas de mudança comportamental, aumentaram a capacidade para caminhar, sendo superiores em proporcionar ganho no desempenho no teste de caminhada de seis minutos na comparação com os exercícios supervisionados realizados em esteira.^13,14^ Ou seja, enquanto programas de exercícios supervisionados em esteira são superiores em aumentar o desempenho da caminhada na esteira, os programas de exercícios baseados em domicílio são superiores para melhorar o desempenho em caminhada no solo, algo mais bem relacionado às atividades da vida diária^14^

Embora em estudos mais recentes os exercícios de caminhada no solo baseados em domicílio tenham se mostrado mais eficazes para o desempenho nas atividades da vida diária, sendo mais indicados do que os exercícios supervisionados em esteira,^14^ os pequenos estudos iniciais mostraram pouco ou nenhum benefício, justificando que nas Diretrizes de Prática Clínica do *American College of Cardiology/American Heart Association* de 2006 constasse equivocadamente “não haver evidências para aconselhar os pacientes a irem para casa caminhar”. Porém, desde 2011 foram publicados ensaios clínicos bem sucedidos que tiveram como intervenção exercícios baseados em domicílio e incluíram substancialmente mais do que conselhos para “ir para casa e caminhar”, solicitando aos pacientes para que estabelecessem metas de exercícios e monitorassem suas atividades físicas, ocasionando a mudança do enfoque. Mesmo com componentes de intervenção comportamental, o exercício baseado em domicílio requer menos recursos e menores custos que o exercício supervisionado em esteira, sendo mais acessíveis e provavelmente mais aceitáveis para muitos pacientes, o que poderia favorecer a uma maior adesão ao tratamento.^13^

Na avaliação funcional de pacientes com DAO de MMII, o teste de caminhada de seis minutos vem ganhando espaço, como uma medida validada, mais bem relacionada aos níveis de atividade física que o teste em esteira, sem estar associado a um efeito de aprendizado quando testes repetidos são realizados.^14^ Em pacientes com DAO de MMII, em comparação aos testes realizados em esteira, as alterações no desempenho observadas na caminhada de seis minutos têm sido melhor associadas a desfechos como mortalidade e perda de mobilidade.^14^

No artigo “Intensidade de Exercício durante o Teste de Caminhada de 6 Minutos em Pacientes com Doença Arterial Periférica”,^1^ o LA foi alcançado no teste de caminhada de 6 minutos em 60% dos indivíduos. O consumo pico de oxigênio obtido durante o teste em esteira, assim como o ITB, foram maiores nos pacientes que não alcançaram o LA durante o teste de caminhada de 6 minutos. O teste de caminhada de 6 minutos foi mais difícil para mulheres e pacientes com baixa aptidão cardiorrespiratória, revelando maior intensidade relativa de esforço de exercício para esses pacientes. Isso é algo relevante na prática, pois visando à melhora da função cardiovascular, recomenda-se que o treinamento físico seja realizado acima do LA.^15^ Portanto, o artigo mostra que existe fundamentação para a utilização de caminhada no solo como modo de exercício preferencial principalmente para mulheres e pacientes com baixa aptidão cardiorrespiratória. Para homens e pacientes com maior aptidão cardiorrespiratória devem ser considerados exercícios mais intensos, em cicloergômetros, ergômetros elípticos ou mesmo esteiras, visando atingir o LA e, consequentemente, melhorar aptidão física.

Por fim, vale mais uma vez ressaltar que o artigo “Exercise Intensity During 6-min Test in Patients with Peripheral Artery Disease” apresenta originais e interessantes resultados, mas sendo um estudo observacional unicêntrico, com pequeno número de indivíduos, obviamente, apresenta limitações que impedem fortes conclusões. Portanto, as importantes informações aplicáveis à prática clínica que constam nos resultados do estudo publicado nesse artigo merecem ser consideradas como temas de futuras pesquisas.
