# Supplementary material for: Exercício Físico e Teste de Caminhada de 6-min na Doença Arterial Obstrutiva de Membros Inferiores
Source: Arq Bras Cardiol. 2020 Apr 6;114(3):493–5. [Article in Portuguese] doi: 10.36660/abc.20200068 (PMC7792735; doi:10.36660/abc.20200068)
Supplement: Translation

# Translation: Exercise and Six-Minute Walk Test in Lower Extremity Occlusive Peripheral Arterial Disease

**Authors:** Tales de Carvalho

**Affiliations:** 1 BrazilClínica de Prevenção e Reabilitação Cardiosport, Florianópolis, SC – Brazil; 2 BrazilUniversidade do Estado de Santa Catarina (UDESC-SC), Florianópolis, SC – Brazil

**Keywords:** Peripheral Arterial Disease, Intermittent Claudication, Anaerobic Threshold, Exercise, Walk Test, Physical Activities of Daily Living

The article “Exercise Intensity During 6-min Test in Patients with Peripheral Artery Disease”^1^ provides original, practically useful information to be considered in the diagnosis, prognosis and mainly in the functional assessment that allows a better prescription of physical exercise in the medical treatment of the disease. The study was conducted with the aim of determining whether overground walking would allows the detection of the first ventilatory threshold, also known as anaerobic threshold (AT), in symptomatic patients with lower extremity occlusive peripheral arterial disease (LE-OPAD). AT is a marker of exercise intensity, useful for the determination of the optimal zone for physical training focused on improvement in cardiorespiratory fitness.^2^

LE-OPAD is an important public health problem. According to global epidemiology report, the disease affected 202 million individuals in 2010, and 237 million in 2015, with a 22% increase during this period.^3^ The association of OPAD with major cardiovascular events (MACE) has been well documented; in the severe stage of the disease, with presence of critical ischemia, there is a high risk of cardiovascular events, lower limb amputation and death,^4^ with association with elevated levels of cardiac troponin and N terminal pro-brain natriuretic peptide (NT-proBNP).^5^

LE-OPAD is highly suspected in the presence of pain in lower limbs when walking, without apparent orthopedic problem, and an ankle brachial index (ABI) lower than 0.90 at rest. Walking tests should be performed to help in the diagnosis, particularly when the ABI is greater than 0.91, and in the functional classification and exercise prescription.^6^Field walking tests allow the identification of intermittent claudication, with determination of the distance walked to symptom onset (initial claudication) and to maximum functional limitation (absolute claudication). In treadmill tests, the measurement of the ABI has been proposed, both at rest and after exercise. The presence of the disease is strongly suspected when ABI is reduced by at least 20% and 30mmHg after exercise compared with rest.^7^ However, resting ABI, which has been widely used in clinical practice, can produce false negative results, which is of particular importance in patients with poorly compressible arteries. In the study by Tóth-Vajna et al.,^8^ almost one fourth of the individuals with a diagnosis had poorly compressible arteries or was considered symptomatic with a negative ABI. Therefore, in case of suspicion of LE-OPAD, a deeper investigation is recommended despite normal ABI values.

In symptomatic patients, exercise can affect morbidity and mortality, with improvement of symptoms and quality of life and increase of maximum walking distance, and thus must be part of the optimized treatment.^6,7^ Therefore, all patients with intermittent claudication should receive optimized medical treatment, i.e., a combination of lifestyle changes with pharmacological therapy, considering the body of evidence showing a reduction in cardiovascular events and improvement of the outcomes related to the lower limbs.^9^

Physical training has been shown to be safe, and the walking tests with claudication symptom induction considered the best option.^6,7^ However, when walking tests cannot be performed, other exercises such as cycling, resistance exercise and exercises using an upper extremity ergometer have been shown effective.^5,6^ It is worth mentioning that patients with critical ischemia cannot perform physical exercises, but should be considered eligible as soon as the interventionist approach is successfully completed.^10,11^

Many clinical trials have consistently shown that supervised treadmill training improves the gait of patients with LE-OPAD. In a meta-analysis, Fakhry et al.^12^ evaluated 1,054 patients from 25 studies and concluded this type of exercise training was effective in increasing maximum walking distance (mean increase of 180 meters) and pain-free walking distance (mean increase of 11 meters).^12^

Three randomized clinical trials that evaluated 493 patients with LE-OPAD, showed that home-based exercise programs that included behavior change techniques, improved walking capacity, and higher performance gain on the six-minute walk test compared with supervised treadmill training.^13,14^In other words, while supervised treadmill walking programs are superior in the improvement of treadmill walking performance, the home-based programs are superior in improving overground walking, which is more related to daily life activities.^14^

Although home-based overground walking programs have been recently shown effective in improving the performance in daily life activities, apart from being more convenient and cheaper compared with supervised treadmill exercise,^14^small older studies showed little or no benefit. Therefore, the American College of Cardiology/American Heart Association 2006 Practice Guidelines state that there was no evidence to support the recommendation for the patient “to go home to walk”. However, since 2011, successful clinical trials with home-based exercise interventions that included much more than recommendations like “go home to walk”, such as the instructions for patients to stablish exercise goals and monitor their exercises, thereby promoting a change of focus. Even adding behavior change techniques, home-based exercises require fewer resources and less cost compared with supervised treadmill exercise, and thus are more accessible and probably more acceptable by many patients, which may ultimately lead to higher treatment compliance.^13^

In functional assessment of patients with LE-OPAD, six-minute walk test has gained popularity as a validated measure and better related to physical activity levels compared with the treadmill test, with no association with the learning effect of repeated tests.^13^ In patients with LE-OPAD, compared with treadmill tests, changes in the performance on six-minute walk test have been more associated with outcomes as mortality and loss of mobility.^14^

In the paper “Exercise Intensity During 6-min Test in Patients with Peripheral Artery Disease”,^1^ AT was reached in 60% of patients, and peak oxygen consumption and ABI during treadmill test were higher in the other 40% patients. The six-minute walk test was more difficult for women and patients with low cardiorespiratory fitness, indicating a higher relative intensity of exercise effort for these patients. This is relevant in practice, since it is recommended that exercise training be performed above the AT aiming at improving cardiovascular function.^15^ Therefore, the study corroborates the use of overground walking as the exercise mode of choice for women and patients with low cardiorespiratory fitness. More intense exercise training should be considered for men and patients with better cardiovascular fitness including cycle and elliptical ergometers, and even treadmills, to reach the AT and consequently improve physical fitness.

Finally, it is worth pointing out that the article “Exercise Intensity During 6-min Test in Patients with Peripheral Artery Disease” provides original and interesting results, but since it is an observational, monocentric study, it has limitations that prevent firm conclusions being drawn. Therefore, the study provides important information that are applicable to clinical practice and should be considered as the subject of future research.
